# Fluticasone furoate/Vilanterol 92/22 μg once-a-day vs Beclomethasone dipropionate/ Formoterol 100/6 μg b.i.d.: a 12-week cost analysis in mild-to-moderate asthma

**DOI:** 10.1186/s40248-016-0055-2

**Published:** 2016-06-06

**Authors:** Roberto W. Dal Negro, Chiara Distante, Luca Bonadiman, Paola Turco, Sergio Iannazzo

**Affiliations:** National Centre for Respiratory Pharmacoeconomics and Pharmacoepidemiology, Verona, Italy; SIHS Health Economics Consulting, Torino, Italy; Research & Clinical Governance, Verona, Italy

**Keywords:** Asthma cost Beclomethasone dipropionate/Formoterol, Bronchial asthma, Cost analysis, Fluticasone furoate/Vilanterol

## Abstract

**Background:**

Asthma is a disease with high cost for the National Health Service. Two of the most recent LABA/ICS combinations for persistent bronchial asthma are Beclomethasone dipropionate/Formoterol (B/F) delivered via the Nexthaler device and Fluticasone furoate/Vilanterol (F/V) delivered via the Ellipta device. No comparison has been carried out yet in terms of cost analysis in asthma, to our knowledge. Aim of the present monocentric, observational, retrospective study was to calculate and compare the costs of mild-to-moderate asthma patients assuming B/F 100/6 μg b.i.d. to those of patients assuming F/V 92/22 μg once-a-day over a 12-week treatment period from the Italian National Health Service perspective.

**Methods:**

Data were obtained automatically and anonymously from the institutional database of the Lung Unit of the Specialist Medical Centre (CEMS), Verona, Italy, UNI EN ISO 9001-2008 validated. FEV_1_ values, number of relapses, healthcare resources as hospitalizations due to asthma relapses, days of hospitalization, general practitioner (GP), specialist visits, and days of inactivity, were recorded over the study period together with the use of extra medications (systemic steroids and antibiotics). In order to compare the outcomes achieved in both groups, the propensity score matching method was used in STATA, and statistical significance was accepted for *p* < 0.05.

**Results:**

Clinical data of 77 patients treated with B/F b.i.d (Group A) and of 40 patients treated with F/V 92/22 μg once-a-day (Group B) were selected. The PS-matching process, designed as matching on the baseline covariates, gender, age, FEV_1_ and comorbidities, returned a cohort of 40 group A patients of the entire cohort matched with 40 patients of group B, fully comparable for demographics and clinical characteristics. In the PS-matched cohort, the mean (±SE) number of relapses per patient during the follow-up was 0.53 (±0.12) in group A and 0.28 (±0.07) in group B. In group A, *n* = 25 (62.50 %), *n* = 9 (22.50 %), and *n* = 6 (15 %) patients had 0, 1, 2 relapses, respectively. In group B, *n* = 29 (72.50 %), and *n* = 11 (27.50 %) had 0 and 1 relapse, respectively. Over the study period, the average number of hospitalizations per patient was 0.15 (±0.06), with 0.28 (±0.12) days of hospitalization in group A, and 0.08 (±0.04) with 0.08 (±0.04) days of hospitalization in group B, respectively. The difference between the two groups in terms of FEV_1_(L) improvement vs baseline was 0.11 in favour of group B (*p* = 0.007). When results were compared, the improvement in lung function obtained in group B proved significantly higher both in terms of absolute FEV_1_ and of FEV_1_ % predicted.

The mean (±SE) cost of hospitalizations per patient was € 345.30 (±133.23) in group A and € 172.65 (±98.18) in group B, respectively, with a mean not significant difference of - € 172.65 in favour of group B (*p* = 0.9). In particular, the mean (±SE) cost for visits per patient was € 26.82 (±3.73) in group A and € 11.36 (±2.30) in group B (*p* = 0.002), and the mean cost for rescue medications per patient was € 35.24 (±6.93) in group A, and € 18.73 (±3.38) in group B, respectively (*p* = 0.05).

**Conclusions:**

Even if both ICS/LABA combinations were checked over a limited period of time, they seem characterized by a different profile in terms of effect on lung function and economic impact on mild-to-moderate asthma. The once-daily inhalation of combined Fluticasone furoate/Vilanterol 92/22 μg showed the potential for enhanced clinical outcomes and reduced costs when compared to Beclomethasone dipropionate/Formoterol 100/6 μg b.i.d.

## Background

Bronchial asthma is a chronic inflammatory disease of the airways. It is characterized by airflow limitation, usually reversible spontaneously or following therapy, bronchial hyper-responsiveness and accelerated decline in lung function, which may progress to irreversible obstruction of the airways [1].

The underlying mechanism responsible for asthma is the excessive presence and activation of inflammatory cells within the mucosal, muscular and vascular structures of the airways, which cause the release of inflammatory mediators and the remodeling of the airways. Clinically it manifests by the appearance and recurrence of cough, dyspnea, wheezing (at rest and/or by physical exertion), and chest tightness [1]. The clinical expression of the disease varies among individuals and in the same patient over time [2].

According to WHO estimates, 235 million people suffer from asthma. The Italian National Institute of Statistics (ISTAT) survey on health and use of health services estimated a prevalence of asthma of 4.2 % (female 4.3 %, male 4.2 %) in Italy in 2012 [3].

The severity of the disease is based on the frequency of symptoms, value of forced expiratory volume in 1 s (FEV_1_), variability of peak expiratory flow (PEF), and Quality of Life. On this basis there are four levels of asthma severity: mild intermittent, mild persistent, moderate persistent and severe persistent.

Asthma is a disease with high cost for the Italian National Health Service. The main costs are indirect and for hospitalization [4]. The total burden of asthma was estimated to be in Italy about 5 billion Euro per year.

Asthma cannot be cured, but appropriate management can control the disorder and enable people to enjoy a good quality of life [2]. The goal of asthma therapy is to achieve and maintain the control of the disease.

The therapeutic strategy includes two main categories of drugs: controller and rescue medications. The controller medications must be taken regularly to keep the disease under control. The rescue medications relieve the acute bronchoconstriction and related symptoms. Since asthma is an inflammatory disease, inhaled corticosteroids (ICS) are the most effective controller medications currently available and represent the first choice of treatment, to which long-acting beta_2_-agonists bronchodilators (LABA) can be added. The combination of these two categories of drugs is the recommended therapeutic strategy for persistent asthma [1].

Two of the most recent LABA/ICS combinations for persistent bronchial asthma are the Fluticasone furoate/Vilanterol (F/V) 92/22 μg delivered via the Ellipta device [5–7] and the Beclomethasone dipropionate/Formoterol 100/6 μg (B/F) delivered via the Nexthaler device [8–10]. While the former combination covers 24 h and is assumed at once-a-day regimen, the latter has to be assumed twice daily (bis in die, b.i.d.).

Although several studies investigated both the effectiveness and the safety of these two ICS/LABA combinations, to our knowledge no cost comparison has been carried out yet.

## Objective

The purpose of the present study was to estimate and compare the costs of mild-to-moderate asthma patients assuming Beclomethasone dipropionate/Formoterol 100/6 μg b.i.d. to those of patients assuming Fluticasone furoate/Vilanterol 92/22 μg once-a-day over a 12-week treatment period from the Italian National Health Service perspective.

## Methods

The study was an observational, retrospective analysis on asthmatic patients referring over the period February-September 2015 to the Lung Unit of the Specialist Medical Centre (CEMS), Verona, Italy.

Data were obtained automatically and anonymously from the institutional, UNI EN ISO 9001-2008 validated database, and the classic Boolean algebraic formulas were used for selections [11]. Selection criteria were: asthma subjects of both genders >18 years of age; non-smoker; with a normal cognitive function; in a stable respiratory condition (spirometrically assessed) in the last 2 weeks before the study start; assuming B/F 100/6 μg b.i.d (Group A) or F/V 92/22 μg once-a-day (Group B) for 12 (±2) weeks. In baseline sex, age, the absolute and the % predicted values of forced expiratory volume in 1 s (FEV_1_ in Litres and FEV_1_ as % predicted), and comorbidities of the patients were recorded. All patients were followed for 12 (±2) weeks. FEV_1_ values, number of relapses, healthcare resources as hospitalizations due to asthma relapses, days of hospitalization, general practitioner (GP), specialist visits, and days of inactivity were recorded over the study period. The adherence to both treatments was recorded and expressed in % inhalations vs expected number of inhalations over the study period. The use of extra medications (systemic steroids and antibiotics) was also recorded.

In order to compare the outcomes of the patients of both groups, the propensity score (PS) matching method [12] was used in STATA [13]. Statistical significance was accepted for *p* < 0.05. This statistical procedure reduces the bias in estimation of treatment effects with observational datasets. The propensity score matching method summarizes pretreatment characteristics of each subject into a single-index variable (the propensity score) that makes the matching feasible. In this study, a logit regression to estimate the propensity score on the baseline covariates age, sex, FEV_1_(%) and presence of comorbidities was used. Furthermore,  the propensity score matching was performed without replacement, i.e. each of 40 patients of the Group B was matched with only one patient of the Group A.

Costs were analysed and reported as mean values per patient over the study period, in terms of: 1) hospitalization costs; 2) visits costs; 3) rescue-medications costs, and 4) main treatment (controller medication) costs.

Hospitalization cost was evaluated as the mean cost for asthma relapse according to national diagnosis-related group (DRG) tariffs [14], weighted for the frequency reported during the 12-week study. The cost of hospitalization due to asthma relapse in the presence of serious comorbid disease was € 2, 537 (DRG 96), while the cost in the absence of serious comorbid disease was €1,832 (DRG 97).

GP visit cost was estimated in €15.17 based on a published cost study inflated to Euro 2015 with the ISTAT consumer price index [15, 16]. The cost for specialist’s visit was €20.66, derived from national inpatient tariffs (Code 89.7) [17].

Cost of steroids and antibiotics (rescue medications) was estimated considering the ex-manufactory pack-prices [18] and the cycles observed of medications. The unit cost of one cycle of steroids and antibiotics was estimated in € 12.40 and € 42.00 respectively, considering only the ex-manufactory pack-prices [18]. The cost of drugs was estimated considering the ex-manufactory pack prices [18] and the adherence to the treatments. The unit price of B/F was € 31.80 for a pack containing an inhaler with 120 doses, and the daily dose was two inhalations/day. The unit price of F/V was € 30.00 for a pack containing an inhaler with 30 doses, and the daily dose was one inhalations/day.

## Results

Clinical data of 77 patients treated with B/F 100/6 μg b.i.d (Group A) and of 40 patients treated with F/V 92/22 μg once-a-day (Group B) were obtained.

The demographics and clinical characteristics of the entire cohort are summarized in Table 1. At the baseline, male prevalence was 33.8 % in group A and 37.5 % in group B. Mean (±SE) age was 51.87 (±1.60) in group A, and 50.18 (±2.43) in group B. Mean (±SE) FEV_1_ in litres (L) was 2.42 (±0.09) in group A, and 2.51 ((±0.12) in group B. Mean (±SE) FEV_1_% pred. was 82.23 % (±1.14) and 81.93 % (±2.00) in group A and B, respectively. Patients with perennial allergy were 61.0 % (47/77) in group A, and 62.5 % (25/40) in group B, while those with seasonal allergy were 39.0 % (30/77) in group A, and 37.5 % (15/40) in group B, respectively. The percentage of patients with established comorbidities was 37.7 % in group A, and 42.5 % in group B. The following comorbid diseases were equally reported in both groups: arterial hypertension, kyphoscoliosis, obesity, severe depression, AIDS, diabetes mellitus, severe osteoporosis and ischemic heart disease. In particular, arterial hypertension was the most prevalent comorbidity in both groups: 12.5 % in group A, and 10.4 % in group B, respectively).

**Table 1:** Baseline characteristics of the entire cohort and of the PS-matched cohort

	Overall cohort			PS-matched cohort		
	Group A	Group B	Difference Group B – Group A	Group A	Group B	Difference Group B – Group A
n	77	40		40	40	
Males (n) (%)	26 (33.8 %)	15 (37.5 %)	-11 (-3.80 %)	15 (37.5 %)	15 (37.5 %)	0
Mean Age (years) (±SE)	51.87 (±1.60)	50.18 (±2.43)	-1.69	49.40 (±2.05)	50.18 (±2.43)	0.78
Mean FEV_1_ % predicted (±SE)	82.23 (±1.14)	81.93 (±2.00)	-0.30	82.40 (±1.63)	81.93 (±2.00)	-0.47
Comorbidities (% of patients)	37.7 %	42.5 %	-4.80 %	42.5 %	42.5 %	0

Forced expiratory volume in 1 s, predicted values (FEV_1_%)

Group A: patients treated with Beclomethasone dipropionate/Formoterol 100/6 μg b.i.d

Group B: patients treated with Fluticasone furoate/Vilanterol 92/22 μg once-a-day

The PS-matching process, designed as matching on the baseline covariates, gender, age, FEV_1_ and comorbidities, returned a cohort of 40 group A patients of the entire cohort matched with 40 patients of group B.

The demographics and clinical characteristics of the PS-matched cohort at the baseline are described in Table 1. The male prevalence in group A was the same as in group B (37.5 %). Mean age (±SE) was 49.40 (±2.05) in group A and 50.18 (±2.43) in group B, respectively. Mean (±SE) FEV_1_% pred. was 82.40 % (±1.63) in group A and 81.93 % (±2.00) in group B. The presence of comorbidities was balanced (42.5 %) in both groups.

In the PS-matched cohort, the mean (±SE) number of relapses per patient during the follow-up was 0.53 (±0.12) in group A and 0.28 (±0.07) in group B. In group A, *n* = 25 (62.50 %), *n* = 9 (22.50 %), and *n* = 6 (15 %) patients had 0, 1, 2 relapses, respectively. In group B, *n* = 29 (72.50 %), and *n* = 11 (27.50 %) had 0 and 1 relapse, respectively (Table 2).

**Table 2:** Visits, hospitalizations, relapses and inactivity days over the 12 weeks

	Overall cohort				PS-matched cohort			
	Group A	Group B	Difference Group B – Group A	*p*	Group A	Group B	Difference Group B – Group A	*p*
n. GP Visits mean (±SE)	0.84 (±0.09)	0.38 (±0.12)	-0.46	<0.001	0.85 (±0.15)	0.38 (±0.12)	-0.47	<0.001
Specialist visits mean (±SE)	0.66 (±0.08)	0.28 (±0.07)	-0.38	<0.001	0.68 (±0.11)	0.28 (±0.07)	-0.40	<0.001
n. Hospitalizations mean (±SE)	0.13 ± 0.04	0.08 ± 0.04	-0.05	0.372	0.15 ± 0.06	0.08 ± 0.04	-0.08	0.11
Days of Hospitalizations mean (±SE)	0.19 ± 0.07	0.08 ± 0.04	-0.12	0.348	0.28 ± 0.12	0.08 ± 0.04	-0.20	0.09
n. Relapses mean (±SE)	0.53 (±0.08)	0.28 (±0.07)	-0.26	0.106	0.53 (±0.12)	0.28 (±0.07)	-0.25	0.12
Days of inactivity mean (±SE)	1.62 (±0.26)	0.68 (±0.20)	-0.95	0.088	1.53 (±0.37)	0.68 (±0.20)	-0.85	0.12

Group A: patients treated with Beclomethasone dipropionate/Formoterol 100/6 μg b.i.d

Group B: patients treated with Fluticasone furoate/Vilanterol 92/22 μg once-a-day

*GP* general practitioner, [*95 % CI* confidence interval], *p* refers to Wilcoxon rank-sum test

Over the study period, the average (±SE) number of hospitalizations per patient was 0.15 (±0.06), with 0.28 (±0.12) days of hospitalization in group A, and 0.08 (±0.04) with 0.08 (±0.04) days of hospitalization in group B, respectively. *n* = 34 patients (85 %) had 0 days of hospitalization; *n* = 3 (7.5 %) had 1 day; *n* = 1 (2.5 %) had 2 days, and *n* = 2 (5 %) patients had 3 days of hospitalization in group A, while *n* = 37 (92.5 %) and *n* = 3 (7.50 %) patients had 0 and 1 day of hospitalization, respectively in group B, (Table 2).

The mean number of cycles of steroids was 0.73 in group A and 0.33 in group B. The mean number of cycles of antibiotics was 0.33 and 0.63 and 0.35 in group A and group B, respectively.

The adherence to the treatments [168 (±14) expected doses with treatment A and 84 (±14) expected doses with treatment B] was 82.2 % in group A and 93.3 % in group B, respectively. In other words, 30 doses were skipped (approximately corresponding to 15 days of treatment) in group A, and to 6 doses were skipped in group B (corresponding to 6 days of treatment).

In order to evaluate the efficacy of B/F and F/V treatments, FEV_1_ in litres (L) and FEV_1_ % predicted values were compared at baseline and at the end of the study period in the PS-matched cohort (Table 3).

**Table 3:** Difference between FEV_1_ values at baseline (T0) and after 12 weeks (T1) in groups A and B

	Group A				Group B				DIfference Group B – Group A	
	T0	T1	Difference T1 – T0	*p*	T0	T1	Difference T1 – T0	*p*-value	Difference T1-T0	*p*-value
FEV_1_ (L) mean (±SE)	2.52 (±0.13)	2.62 (±0.12)	0.10	0.519	2.51 (±0.12)	2.72 (±0.13)	0.21	0.229	0.11	0.007
FEV_1_ (%), mean (±SE)	82.40 (±1.63)	87.08 (±1.58)	4.68	0.043	81.93 (±2.00)	89.50 (±2.64)	7.57	0.009	2.89	0.04

T0 =at baseline, before Beclomethasone dipropionate/Formoterol and Fluticasone furoate/Vilanterol treatment

T1 = after 12 weeks of Beclomethasone dipropionate/Formoterol and Fluticasone furoate/Vilanterol treatment

Forced expiratory volume in 1 s, litres, FEV_1_ (L)

Forced expiratory volume in 1 s, predicted values, FEV_1_(%)

[95 % CI, confidence interval]

*p* refers to Wilcoxon rank-sum test

Group A: patients treated with Beclomethasone dipropionate/Formoterol 100/6 μg b.i.d

Group B: patients treated with Fluticasone furoate/Vilanterol 92/22 μg once-a-day

In group A mean (±SE) FEV_1_ (L) value was 2.52 (±0.13) at baseline and 2.62 (±0.12) at the end of the study, and the difference vs baseline was 0.10. In this group, mean FEV_1_ % pred. was 82.40 % (±1.63) and 87.08 % (±1.58) at baseline and at the end of the study, respectively; the FEV_1_ % pred. difference vs baseline was 4.68 %.

In group B the mean (±SE) FEV_1_ (L) was 2.51 (±0.12) at baseline and 2.72 (±0.13) at the end of the study. The FEV_1_ difference vs baseline was 0.21. In this group, mean (±SE) FEV_1_ % pred. was 81.93 % (±2.00) and 89.50 % (±2.64) at baseline and at the end of the study, respectively; the FEV_1_ % pred. difference vs baseline was 7.57 %.

The difference between the two groups in terms of FEV_1_ (L) improvement vs baseline was 0.11 in favour of group B (*p* = 0.007). The corresponding difference in terms of FEV_1_ % pred. improvement vs baseline was 2.89 in favour of group B (*p* = 0.04).

In the PS-matched cohort, the mean (±SE) cost of hospitalizations per patient was € 345.30 (±133.23) in group A and € 172.65 (±98.18) in group B, respectively, with a mean difference of - € 172.65 in favour of group B. The mean (±SE) cost for GP visits per patient was € 12.88(±2.27) in group A and € 5.68(±1.77) in group B, with a mean difference - € 7.20 in favour of group B. The mean (±SE) cost for specialist visits per patient was € 13.95 (±2.27) in group A and € 5.68 (±1.48) in group B, with a mean difference - € 8.26 (Table 4).

**Table 4:** Estimate of the resource costs for the entire cohort and of the PS-matched cohort over the study period (€/per patient/12 weeks)

	Overall cohort (Euro)				PS-matched cohort (Euro)			
	Group A	Group B	Difference Group B – Group A	*p*	Group A	Group B	Difference Group B – Group A	*p*
Cost of GP visits mean (±SE)	12.79 (±1.43)	5.68 (±1.77)	-7.11	<0.001	12.88 (±2.27)	5.68 (±1.77)	-7.20	<0.001
Cost of specialist visits mean (±SE)	13.68 (±1.60)	5.68 (±1.48)	-8.00	<0.001	13.95 (±2.27)	5.68 (±1.48)	-8.26	0.002
Cost of hospitalization mean (±SE)	274.55 (±82.76)	172.65 (±98.18)	-101.90	0.396	345.30 (±133.23)	172.65 (±98.18)	-172.65	0.98

Group A: patients treated with Beclomethasone dipropionate/Formoterol 100/6 μg b.i.d

Group B: patients treated with Fluticasone furoate/Vilanterol 92/22 μg once-a-day

*GP* general practitioner, [*95 % CI* confidence interval], *p* refers to Wilcoxon rank-sum test

Moreover, the mean cost for rescue medications per patient was € 35.24 in group A, and € 18.73 in group B, respectively, with a difference of - € 16.51 in favour of group B. The mean costs of the treatments per patient considering the ex-manufactory pack prices and the adherence were € 36.60 in group A, and € 78.37 in group B. Overall, the mean total cost per patient during the study period was € 443.97 in group A, and € 281.11 in group B, with a difference of - €162.86 in favor of group B.

No relevant side effect was reported in both groups of patients. Transient hoarseness was recorded in two patients in group B and in one patient of group A, while transient tachycardia was recorded in two patients of group A.

## Discussion

Asthma is a disease characterized by variable degree of airway obstruction which is mainly related to variable degree of airway inflammation. In persistent mild-to-moderate asthma a therapeutic strategy based on regular assumption of ICS, or ICS/LABA is recommended in order to prevent and/or avoid the occurrence of asthma relapses.

Several factors can affect the results of different treatments, such as: the pharmacological characteristics of the prescribed molecules; the handling characteristics of the inhaler devices used for the drug delivery; the required frequency of daily inhalations for a 24 h efficacy of treatment(s); the patient’s adherence to treatment. Also the cost of treatments is progressively regarded as a factor which can significantly contribute to precise the economic profile of different treatments, and then to compare their convenience, particularly from the National Health Service perspective.

The present study was an observational, retrospective analysis aimed to compare the costs of mild-to-moderate asthmatic patients assuming Fluticasone furoate/Vilanterol once-a-day or Beclomethasone dipropionate/Formoterol b.i.d over a 12-week period.

Both treatments were effective in improving lung function significantly, particularly when the difference calculated from baseline was measured as FEV_1_ % predicted. When the results obtained in group A and B were compared, the improvement obtained in group B proved significantly higher both in terms of absolute FEV_1_ and FEV_1_ % predicted.

The trend in favour of treatment B was also confirmed by the cost analysis.

In general terms, the cost analysis showed a tendency towards a clear reduction of mean total costs in patients treated with Fluticasone furoate/Vilanterol 92/22 μg once-a-day. The hospitalization cost, which is the main component of cost, dropped by 50 % with Fluticasone furoate/Vilanterol 92/22 μg once-a-day. Unfortunately, due to both a certain dispersion of data occurring during the limited duration of the study in a small sample of subjects, and to the low frequency of hospitalization events over this short period of time, the comparison between the two groups in terms of hospitalization cost did not reach the statistical significance.

In particular, even calculated over this limited period of investigation, the number of GP and Specialist consultations was significantly lower in patients treated with Fluticasone furoate/Vilanterol. Correspondingly, the cost due to visits (i.e. GP and Specialist visits) and to extra-medications (rescue medications) needed for facing relapses (such as, oral steroids and antibiotics) were also significantly lower in this group, thus confirming the substantial decrease of asthma impact during Fluticasone furoate/Vilanterol treatment.

Even if both ICS/LABA combined treatments are regarded as highly effective and safe in persistent asthma [5–10], nonetheless they seem characterized by a different profile in terms of their economic impact on mild-to-moderate asthma patients. On the other hand, Formoterol and Vilanterol are characterized by different pharmacokinetics and pharmacodinamics, as well as Beclomethasone dipropionate and Fluticasone furoate are [19–21]. The corresponding fixed combinations obviously reflect these pharmacological peculiarities that lead to different clinical aspect of their action and utilization, such as the higher selectivity and persistency on steroid receptors in favour of Fluticasone furoate, together with the higher selectivity and a persistency on ß_2_- receptors in favour of Vilanterol. Basically, these peculiarities are those which consent the 24-h therapeutic effect of the Fluticasone furoate/Vilanterol combination and then the once-a-day assumption, which likely leads to a better patients’ adherence to a long-term therapeutic strategy as confirmed in the present paper over a 12-week period.

The present study has some limits: the short duration of the investigation (12 weeks only) and the relative small number of subjects included. Moreover, the study is a monocentric investigation. On the other hand, some points of strength consist in the automatic selection of subjects from a general data-base and in the use of the propensity score matching method for assessing and comparing the outcomes, which assured a strictly objective system for comparison between the two subjects’ samples.

## Conclusions

The present study showed that the once-daily inhalation of combined Fluticasone furoate/Vilanterol 92/22 μg has the clear potential for enhanced clinical outcomes and reduced costs when compared to Beclomethasone dipropionate/Formoterol 100/6 μg b.i.d.
